# Anxiety and depression symptoms during the COVID-19 pandemic in European countries and Australia

**DOI:** 10.1093/eurpub/ckac129.744

**Published:** 2022-10-25

**Authors:** K Gemes, J Bergström, M Sijbrandij, I Pinucci, S Quero, J van der Waerden, S Burchert, RA Bryant, E Mittendorfer-Rutz

**Affiliations:** 1 Department of Clinical Neuroscience, Karolinska Institutet, Stockholm, Sweden; 2 Department of Clinical, Neuro- and Developmental Psychology, Amsterdam Public Health Institute, Vrije Universit, Amsterdam, Netherlands; 3 Department of Human Neurosciences, Sapienza University of Rome, Rome, Italy; 4 Department of Basic, Clinical Psychology and Psychobiology, Universitat Jaume I, Castellón, Spain; 5 CIBER de Fisiopatología de la Obesidad y Nutrición, Carlos III Institute of Health, Madrid, Spain; 6 INSERM U1136, Sorbonne Université, Institut Pierre Louis d'Épidé, Paris, France; 7 Department of Education and Psychology, Freie Universität Berlin, Berlin, Germany; 8 University of New South Wales, Sydney, Australia

## Abstract

**Background:**

Studies on mental health changes during the COVID-19 pandemic report no change or increasing prevalence of mental health problems in general, but less is known on changes in potentially disadvantaged groups over time. We investigated changes in anxiety and depression symptoms during the first year of the pandemic in France, Germany, Italy, the Netherlands, Spain, Sweden, and Australia by prior mental disorders and migration status.

**Methods:**

Overall, 4,674 adults answered a web-based survey in May-June 2020 and were followed by three repeated surveys up to February 2021 in these countries. Information on socio-demographic, living conditions, psychosocial factors, diagnosis of mental disorders before, depression and anxiety symptoms during the pandemic and migration status (being a resident or not) was collected. Weighted general estimation equations modelling was used to investigate the association between prior mental disorders, migration status, and symptoms over time.

**Results:**

Most participants were <40 years old (48%), women (78%), and highly educated (62%) with some variations across countries. The baseline prevalence of depressive and anxiety symptoms ranged between 19%-45% and 13%-35% respectively. In most countries, prevalence remained unchanged throughout the pandemic and was higher among people with prior mental disorder than without even after adjustment for socioeconomic, psychosocial, living and health factors. We observed interactions between previous mental disorders and symptoms of anxiety or depression over time in Germany (p = 0.01) and in Spain (p = 0.04). No prevalence difference was noted by migration status.

**Conclusions:**

Depression and anxiety symptoms were worse among individuals with prior mental disorders than without, but there was no clear trend of mental health worsening in the observed groups during the first year of the pandemic. Still, monitoring mental health should be continued in the long-term, with special focus on vulnerable groups.

**Key messages:**

• Depression and anxiety symptoms were higher in individuals with prior mental disorders during the first year of the pandemic in an international sample of six European countries and Australia.

• There were no clear trends of mental health worsening in any of the observed groups in neither of the countries between May-June 2020 and February 2021.

